# An Anatomic Study of the Lingual Nerve and Associated Branches

**DOI:** 10.1002/cre2.70051

**Published:** 2025-02-05

**Authors:** René Human‐Baron, Alexander Procos, André Uys

**Affiliations:** ^1^ Department of Anatomy, School of Medicine, Faculty of Health Sciences University of Pretoria Hatfield South Africa

**Keywords:** lingual nerve, mandibular molars, submandibular duct

## Abstract

**Objectives:**

The aim of the study was to investigate the course and anatomy of the lingual nerve (LN) to minimize the risk of iatrogenic damage during dental procedures.

**Material and Methods:**

The LN was dissected in 25 cadavers (dentulous and edentulous). The intersection of the LN and submandibular duct (SMD), the bifurcation location from the mandibular nerve, the branching patterns of the main trunk, and the number of terminal branches were recorded.

**Results:**

In dentulous females, LN distances to the third, second, and first molars were 11.46 ± 2.51, 15.50 ± 5.24, and 18.75 ± 5.91 mm, while in dentulous males, they were 10.98 ± 1.27, 15.75 ± 2.61, and 19.65 ± 4.50 mm, respectively. For edentulous mandibles, all distances were shorter compared to the dentulous group. In 39.13%, the LN passed superior to the SMD. The LN is bifurcated above the mandibular notch in all cases. The number of branches entering the tongue ranged from 2 to 9, with a Type 1 branching pattern found to be the most prevalent.

**Conclusions:**

The study incorporated both dentulous and edentulous cadavers from a South African population. The findings hold significance for surgical procedures, providing valuable insights into minimizing potential damage.

## 1 Introduction

The risk of lingual nerve (LN) damage rises during procedures in the oral cavity near the mandibular third molar due to the intricate network of branches, distributions, and connections in this area (Erdogmus, Govsa, and Celik 2008). The LN is at risk of injury during dental procedures such as extractions, inferior alveolar nerve blocks, tumor resection, and reconstructive surgery (Tojyo et al. 2019). Compression of the LN can lead to sensory deficits or neuropathic pain syndromes (Graff‐Radford and Evans 2003). The estimated rate of permanent LN injury during third molar removal surgery ranges from 0.02% to 2% (Tojyo et al. 2019). According to Tojyo et al. (2019), iatrogenic LN injuries are found to be higher in patients with disto‐angular positioned third molars (Tojyo et al. 2019). Disto‐angular molars decrease the distance between the LN and the third mandibular molar, resulting in an increased rate of injury (Tojyo et al. 2019). The crown of the molar may also be anatomically close to the LN, resulting in an increased risk of injury (Tojyo et al. 2019). Periodontal and implant surgeries require the location of the LN to prevent injuring the LN, especially in relation to the first and second mandibular molar (Chan et al. 2010). Injury to the LN can also induce changes in the tongue's epithelium due to its association with the chorda tympani (Piagkou et al. 2011). LN injury can result in taste changes, altered sensation, speech difficulties, increased pain, swallowing difficulties, reduced liquid and food control, and a burning sensation of the tongue (Kushnerev and Yates 2015; Pichler and Beirne 2001; Bagheri et al. 2009).

The LN is a branch of the posterior trunk of the mandibular nerve and carries general visceral afferent fibers (Erdogmus, Govsa, and Celik 2008). The bifurcation of the LN from the mandibular nerve occurs approximately 8.7 ± 4.2 mm below the foramen ovale (Shinohara, Mataga, and Kageyama 2010). In contrast Kim et al. (2003) identified the bifurcation point 14.3 mm inferior to the foramen ovale. In their study, Kim et al. (2003), observed that the LN bifurcated above the mandibular notch in approximately 65.6% of cases. Additionally, 25% of cases demonstrated bifurcation in the upper half between the mandibular notch and the mandibular lingula, while 3.1% of cases showed bifurcation in the lower half between the mandibular notch and the mandibular lingula (Kim et al. 2003).

**Figure 1:**
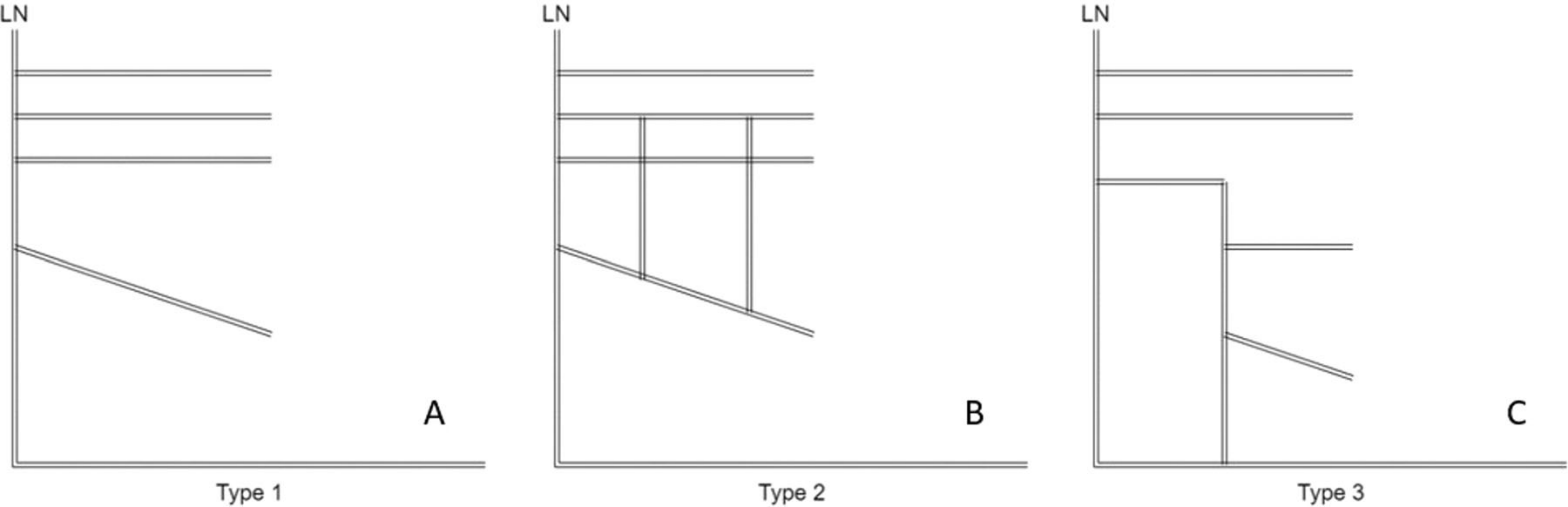
Type of LN branching patterns. (A) Type 1 branching pattern, (B) Type 2 branching pattern, and (C) Type 3 branching pattern (Shimotakahara et al. 2019). LN = lingual nerve.

The descending LN travels between the lateral pterygoid and the tensor veli palatini muscles and receives, at this position, a branch from the facial nerve, the chorda tympani nerve (Kim et al. 2003; Pogrel et al. 1995). The main trunk gives off several branches (Shimotakahara et al. 2019). A study by Shimotakahara et al. (2019), categorized the branches into three main types (Shimotakahara et al. 2019). A Type 1 branching pattern occurs when the LN branches at nearly equal intervals. Type 2 is similar to Type 1 but includes communicating branches. Type 3 features a large branch that further subdivides into additional branches (Figure 1). Understanding the locations of these branches can enhance the outcomes of oral surgical procedures.

The LN travels close to the junction between the body and the ramus of the mandible on its medial surface, passing medially to the roots of the third molar and through the submandibular region (Erdogmus, Govsa, and Celik 2008; Al‐Amery et al. 2016; Sittitavornwong et al. 2017). A study by Ostrowski et al. (2023) measured the vertical distances between the lingual nerve (LN) and the first, second, and third mandibular molars using cadavers, MRI, ultrasound, and surgical methods. The distances were found to be 19.81 ± 5.41, 14.62 ± 3.22, and 11.59 ± 2.12 mm, respectively. Mendes et al. (2013) conducted horizontal and vertical measurements to determine the distance between the alveolar ridge and the lingual nerve (LN). By dissecting cadavers after sagittal sectioning of the heads, they found that the horizontal distance between the LN and the alveolar ridge of the third molar was 4.4 ± 2.4 mm. The vertical distance between the LN and the alveolar ridge near the third molar was 16.8 ± 5.7 mm. A study conducted by Chan et al. (2010) measured the vertical distance from the first and second molars to the lingual nerve (LN). The researchers found that the distance between the LN and the second molar was 9.5 ± 3.9 mm on the right side and 9.7 ± 2.9 mm on the left side (Chan et al. 2010). For the first molar, Chan et al. (2010) found the distance to be 12.7 ± 3.7 mm on the right and 13.2 ± 4.3 mm on the left side. Chalise et al. (2022) measured the distance from the LN to the third mandibular molar tooth in a sample of 15 male cadavers and found the mean vertical distance to be 12.67 ± 1.76 mm (Chalise et al. 2022).

The LN travels anteriorly, in between the submandibular gland, and crosses the submandibular duct (SMD). In a study by Mendes et al. (2013), it was found that in 62.5% of cases, the LN traveled inferior to the SMD, while the remaining 37.5% traveled superior to the SMD (Al‐Amery et al. 2016; Mendes, de Carvalho Leite Leal Nunes, and de Almeida Lopes 2013). In a recent study by Iwai et al. (2022), the LN was found inferior to the SMD 99.4% of the time, while only 0.6% of SMD was found above the LN. Their sample size was 1436 LNs (Iwai et al. 2022). Ostrowski et al. (2023) found that the LN traveled inferiorly to the SMD 68.39% of the time, while 33.14% traveled superiorly to the SMD.

After crossing the SMD, the LN enters the tongue on its ventral surface, anterior to the circumvallate papillae, and continues anteriorly toward the apex of the tongue (Zur, Mu, and Sanders 2004). The LN gives general sensory innervation of the anterior two‐thirds of the lateral aspect of the tongue, while the central region has minimal LN branches (Al‐Amery et al. 2016; Zur, Mu, and Sanders 2004). The gingiva near the roots of the third molar, the floor of the mouth, and the anterior two‐thirds of the tongue are innervated by the collateral nerve twigs of the LN (Kim et al. 2003; Zur, Mu, and Sanders 2004).

According to Al‐Amery et al. (2016), the LN enters the tongue in a varying degree of patterns (Al‐Amery et al. 2016). Pattern 1 has only a single terminal branch from the LN; pattern 2 has two terminal branches; pattern 3 has three terminal branches; and pattern 4 has 4 or more terminal branches (Al‐Amery et al. 2016). Pattern 1 was found to be the least common, pattern 2 was the most common, comprising half of the sample, pattern 3 had a prevalence of 28.6%, and pattern 4 had a prevalence of 14.3%. Zur et al. (2004) found that the LN has a medial and lateral branch. The medial branch gave off 2–4 branches while the lateral branch gave off 3–4 branches (Zur, Mu, and Sanders 2004).

While there has been some exploration of the course, distribution, and anastomosis of the LN in previous studies (Erdogmus, Govsa, and Celik 2008; Sittitavornwong et al. 2017; Zur, Mu, and Sanders 2004; Touré et al. 2005; Rusu et al. 2008; Shimoo et al. 2017), further investigation is needed to fully understand its associated branches, branching patterns and course. Thus, the purpose of this study is to acquire morphological data regarding the distance of the LN to the mandibular molars, its relation to the SMD, bifurcation point, number of terminal branches entering the tongue, and the branching pattern of the main trunk of the LN.

## 2 Materials and Methods

This study was an observational cross‐sectional study conducted in the Department of Anatomy, University of Pretoria, Faculty of Health Sciences. Ethical clearance for the use of cadaver tissue was obtained from the Faculty of Health Sciences Research Ethics Committee (185/2023). All dissections, measurements, and observations were in accordance with the Declaration of Helsinki. Informed consent for the use of the cadavers was not needed as the Department of Anatomy, University of Pretoria, is the custodian of all the cadaver material.

Access to the oral cavity was gained by hemi‐sectioning of the cadaver heads using an oscillating saw.

Twenty‐five white South African embalmed cadavers with intact mandibles were dissected bilaterally, resulting in a sample size of 50 nerves. The sample consisted of 16 males and nine females with an average age of 76.2 ± 7.93 (62–102) years. Six females and five males were dentulous, resulting in a dentulous sample size of 11. Three females and 11 males were edentulous, resulting in an edentulous sample size of 14.

Only cadavers with an intact mandible and complete LN were included in this study. Any cadavers with deformities, pathology, previous surgeries to the mandible, or fractured mandibles were excluded from this study. After the exclusion criteria were implemented, a sample of 50 lingual nerves (LNs) was used for the shortest distance from the LN to the third molar, and 49 LNs were used for the shortest distance from the LN to the second and first molar as one of the LNs were damaged. A total of 46 LNs were used for the relationship with the SMD, as some SMDs were damaged. Determining the level at which the LN bifurcated was done on a sample of 49 cadavers, as the mandibular nerve was damaged in one cadaver. There were 48 LNs observed that entered the tongue (the branches of the 48 LNs that entered the tongue had a sample size of 48) since some of the branches were damaged. One LN was damaged; therefore, 49 LNs were observed for the branching pattern.

**Figure 2:**
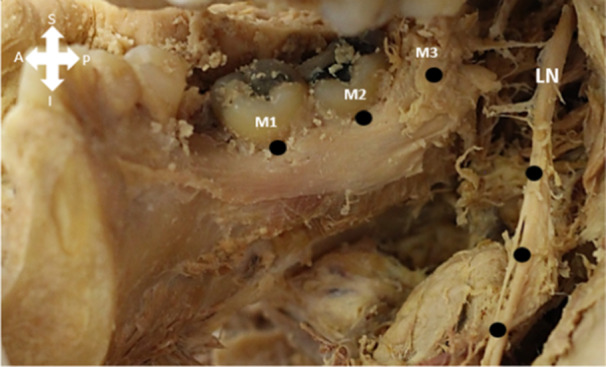
The points represent the locations where the distances were measured for the first (M1), second (M2), and third (M3) mandibular molar to the lingual nerve (LN). Please note that the LN was moved medially to show the measuring points and not the true distance. S = superior; I = inferior; A = anterior; P = posterior.

The area medial to the third molar was dissected to identify the LN. The LN was followed anteriorly to its entry into the tongue. The shortest distance between the first, second, and third molar, and the LN was measured with a sliding caliper (to the closest mm). Dentulous mandibles were measured from the lingual aspect of the alveolar ridge of each molar to the closest aspect of the LN for both the right and left sides. For edentulous mandibles, the distance between the lingual aspect of the mandible in close relation to the lingual aspect of the alveolar crest of each molar tooth was measured (Figure 2). The middle of each molar socket on the medial side of the mandible was used.

**Figure 3:**
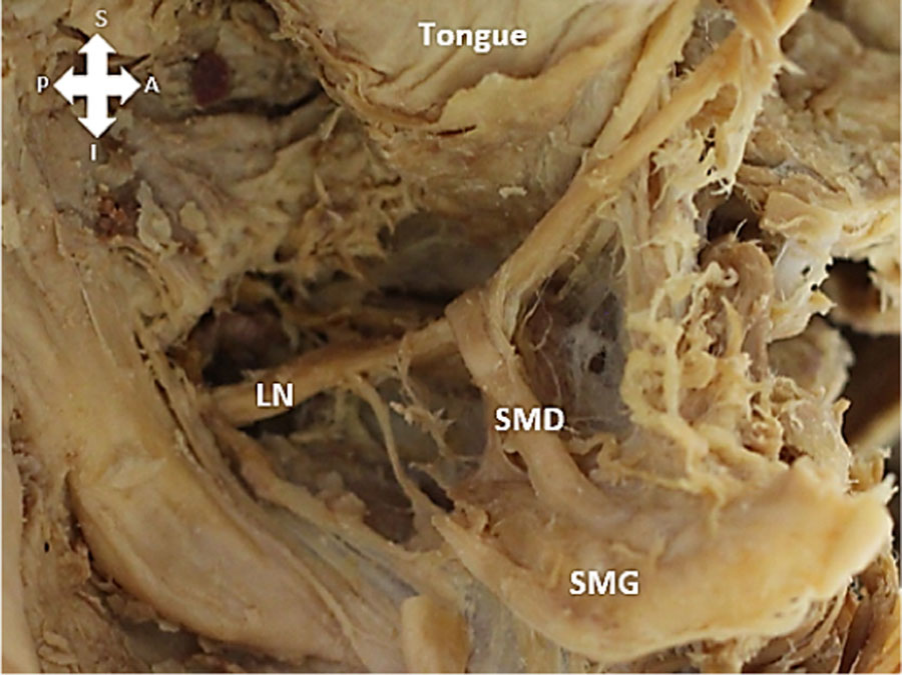
The lingual nerve (LN) traveling beneath the SMD. SMD = submandibular duct, SMG = submandibular gland, S = superior, I = inferior, A = anterior, P = posterior.

The course of the LN in relation to SMD was noted as either passing superior or inferior to the SMD before entering the tongue (Figure 3).

**Figure 4:**
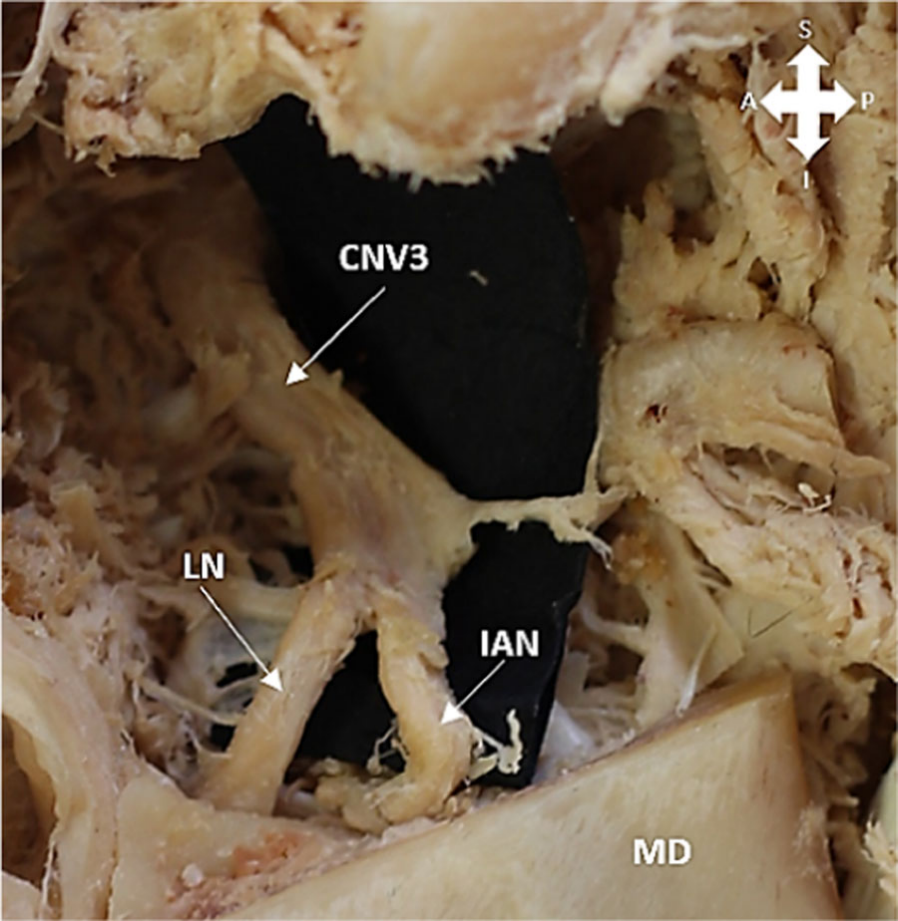
Bifurcation of the lingual nerve (LN) and the inferior alveolar nerve (IAN) from the mandibular division of the trigeminal nerve (CNV3). MD = Mandible, S = superior, I = inferior, A = anterior, P = posterior.

The LN's bifurcation after it branched from the mandibular nerve was observed and recorded in relation to the mandibular notch, upper or lower half between the mandibular notch and the mandibular lingula, or at the mandibular lingula (Figure 4).

**Figure 5:**
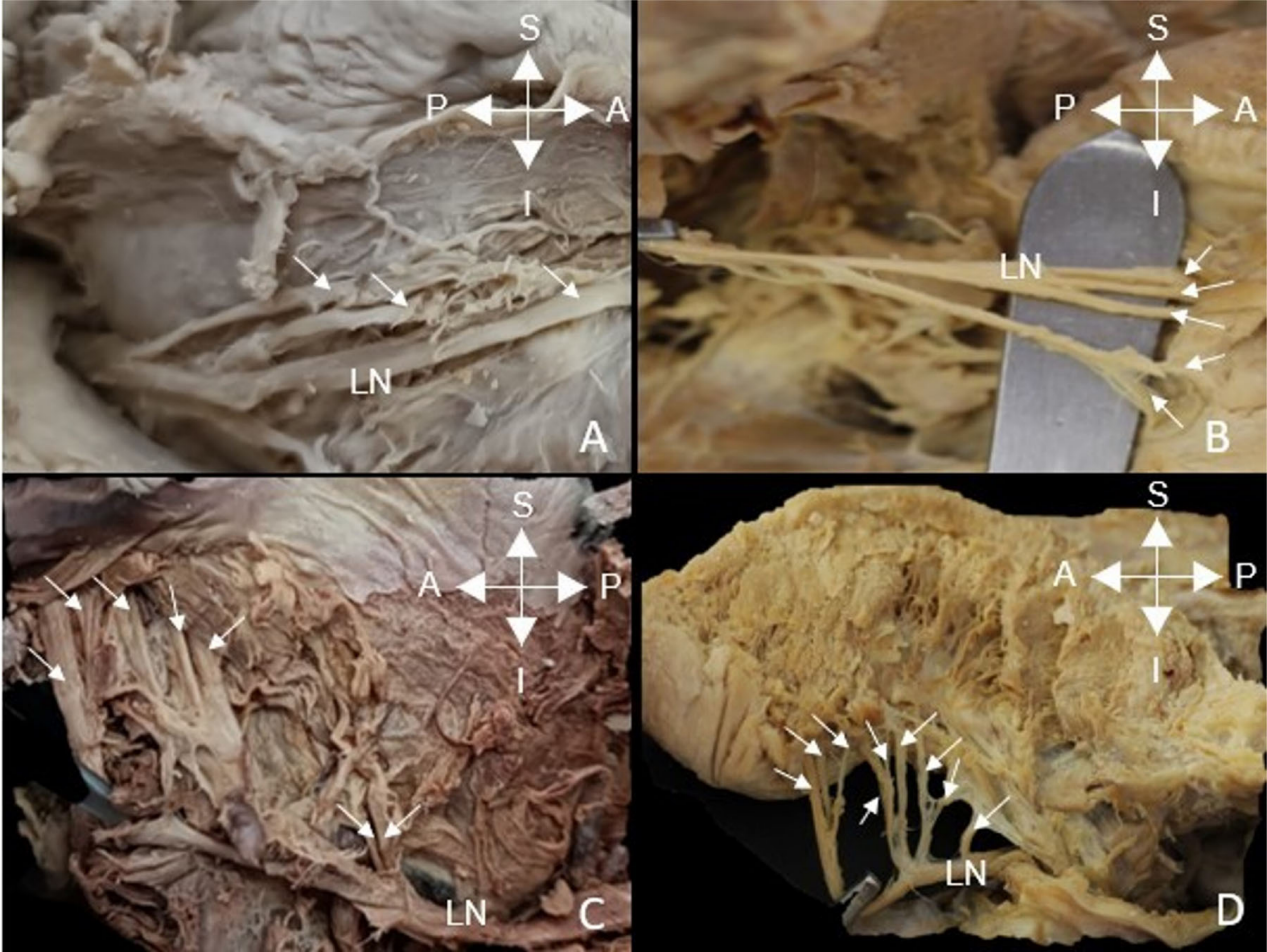
Lingual nerve (LN) branches that enter the tongue: (A) 3 terminal branches, (B) 5 terminal branches, (C) 7 terminal branches, and (D) 9 terminal branches. S = superior, I = inferior, A = anterior, P = posterior.

The number of branches and the configuration of the LN entering the tongue were recorded (Figure 5).

**Figure 6:**
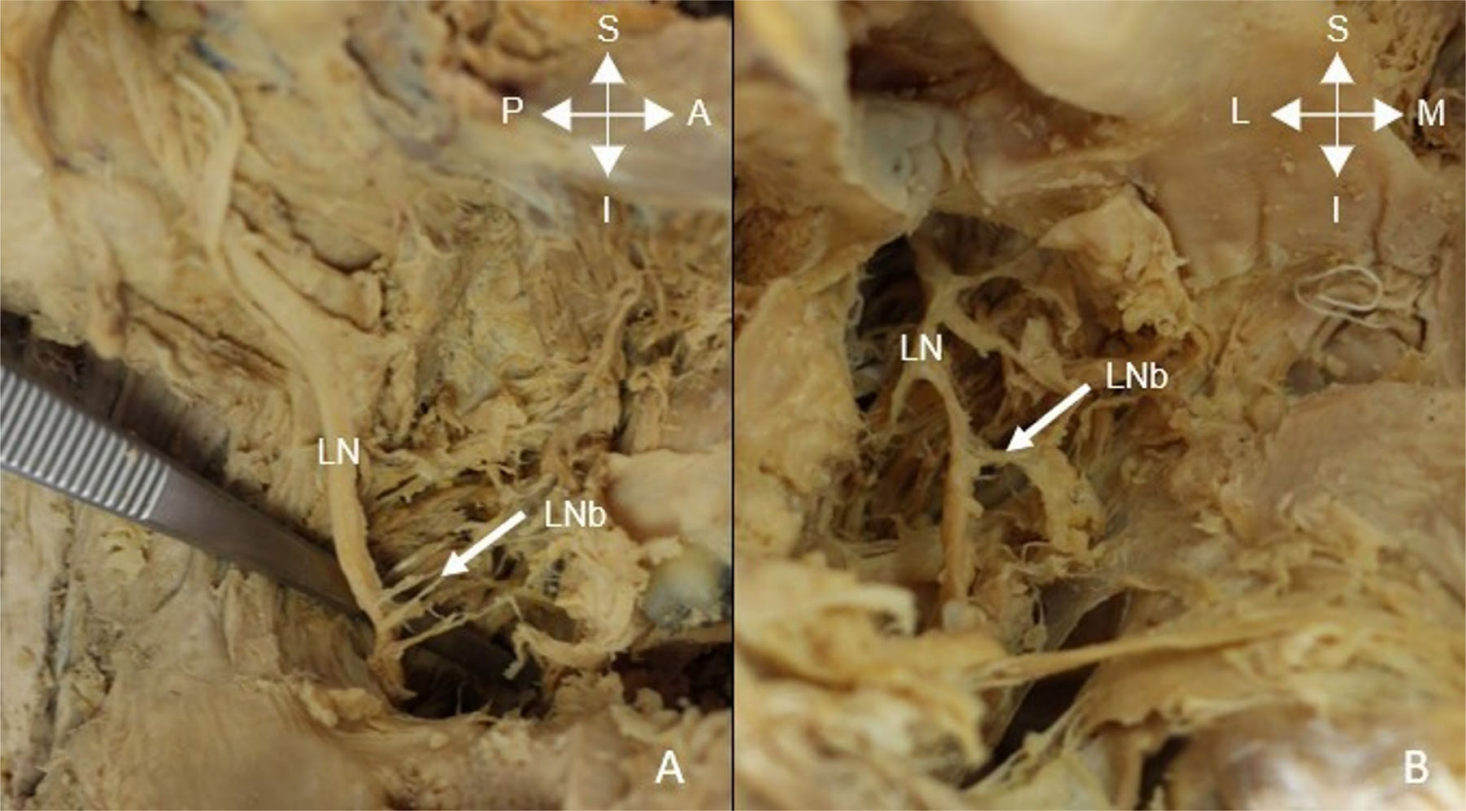
The types of lingual nerve (LN) branches. (A) Type 1 branching pattern and (B) Type 2 branching pattern. LNb = lingual nerve branches, L = lateral, M = medial, S = superior, I = inferior, A = anterior, P = posterior.

After the main trunk of the LN was given off, the branching pattern was noted as either a Type 1, 2, or 3 as described in Figure 1. Figure 6 shows a Type 1 branching pattern.

Demographic data such as age, sex, side, and population group were reported using descriptive statistics. The mean, median, standard deviation, minimum, and maximum of each measurement to the mandibular first, second, and third molar were calculated. Frequencies of the relation of the LN to the SMD, bifurcation pattern, terminal branches entering the tongue, and branching pattern of the main trunk of the LN were recorded.

## 3 Results

Intra‐observer reliability for the measurements and observations was assessed by repeating each measurement three times, calculating the average of the three measurements, and then determining the percentages of the total.

**Figure 7:**
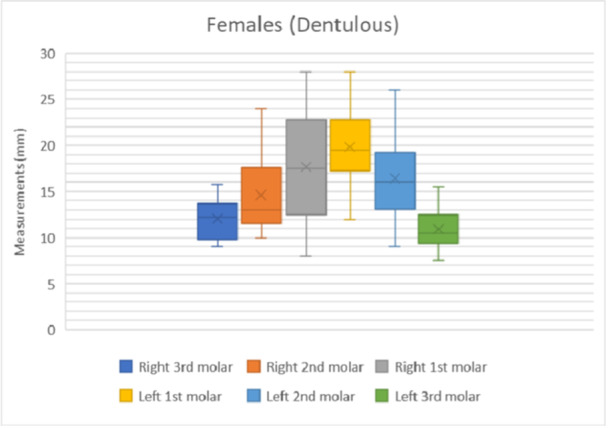
Distance to LN from each mandibular molar in dentulous females. x = mean average of all data points, solid horizontal line = median value.

**Figure 8:**
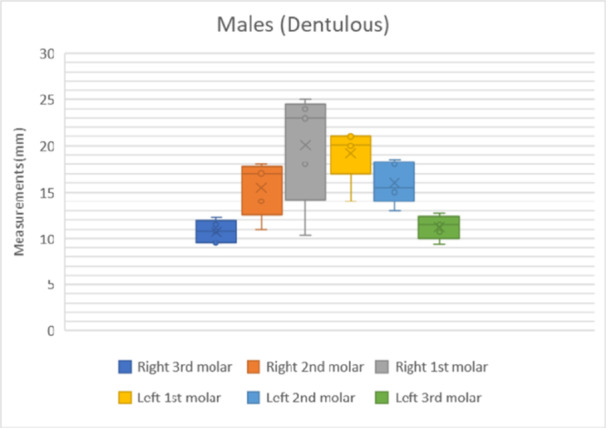
Distance to LN from each mandibular molar in dentulous males. x = mean average of all data points, solid horizontal line = median value.

**Figure 9:**
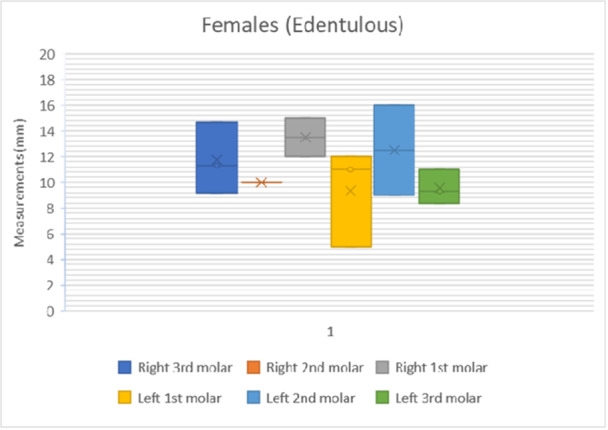
Distance to LN from each mandibular molar in edentulous females. x = mean average of all data points, solid horizontal line = median value.

**Figure 10:**
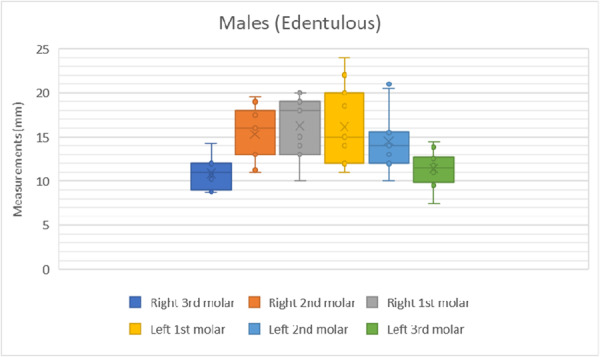
Distance to LN from each mandibular molar in edentulous males. x = mean average of all data points, solid horizontal line = median value.

The results of the shortest distance to the LN from each mandibular molar is shown in Figures 7, 8, 9, 10. The data were separated into dentulous and edentulous groups for males and females.

The distances from the first, second, and third molars for both sexes and sides of the dentulous and edentulous cadavers are given in Table 1. This includes the minimum and maximum distances (Figures 7, 8, 9, 10). In dentulous males, the shortest distances between the lingual nerve and the right third, second, and first molars were 9.50, 11.00, and 10.40 mm, respectively, with maximum distances of 12.33, 18.00, and 25.00 mm. The mean distances were 10.73, 15.50, and 20.08 mm. For dentulous females, the shortest distances were 9.00, 10.00, and 8.00 mm, with maximum distances of 15.83, 24.00, and 28.00 mm. The mean distances were 12.03, 14.58, and 17.67 mm. In edentulous males, the shortest right side distances were 8.67, 11.00, and 10.00 mm, with maximums of 14.17, 19.50, and 20.00 mm. The female edentulous group had shortest right side distances of 9.17, 10.00, and 12.00 mm, with maximums of 14.67, 11.00, and 15.00 mm. For edentulous males, the shortest left‐side distances were 11.00, 10.00, and 7.50 mm, while females had 5.00, 9.00, and 8.33 mm.

**Table 1:** The minimum, maximum, and mean distance of the lingual nerve to the first, second, and third molars in dentulous and edentulous male and female cadavers.

Statistics	Right third mandibular molar (mm)	Right second mandibular molar (mm)	Right first mandibular molar (mm)	Left first mandibular molar (mm)	Left second mandibular molar (mm)	Left third mandibular molar (mm)
Dentulous						
Females						
Min	9	10	8	12	9	7.5
Max	15.83	24	28	28	26	15.5
Mean	12.03	14.58	17.67	19.83	16.42	10.89
SD	2.4	4.94	6.71	5.12	5.54	2.63
Males						
Min	9.5	11	10.4	14	13	9.33
Max	12.33	18	25	21	18.5	12.67
Mean	10.73	15.5	20.08	19.22	16	11.23
SD	1.24	2.96	6.04	2.96	2.26	1.29
Edentulous						
Females						
Min	9.17	10	12	5	9	8.33
Max	14.67	11	15	12	16	11
Mean	11.72	10.5	13.5	9.33	8.33	9.56
SD	2.77	0.71	2.12	3.79	4.95	1.35
Males						
Min	8.67	11	10	11	10	7.5
Max	14.17	19.5	20	24	21	14.5
Mean	10.86	15.29	16.27	16.14	14.5	11.44
SD	1.65	3.07	3.41	4.35	3.42	2

*Note:* Minimum (Min), maximum (Max); mean and standard deviation (SD).

The average distances from the mandibular molars to the LN were recorded (Table 2) for dentulous females and males and edentulous females and males. The average distance between the first, second, and third mandibular molars and lingual nerve for the male dentulous group was 19.65 ± 4.50, 15.75 ± 2.61, and 10.98 ± 1.27 mm. For the female dentulous group, the average distances between the first, second, and third molars and lingual nerve were 18.75 ± 5.91, 15.50 ± 5.24 mm, and 11.46 ± 2.51 mm, respectively. The grouped average for the distance between the first, second, and third molars and lingual nerve for the dentulous males and females was 19.20 ± 5.21, 15.63 ± 3.93, and 11.22 ± 1.89 mm, respectively. The averages for the edentulous group for the males for the distance between the first, second, and third molars were 16.20 ± 3.88, 14.90 ± 3.25, and 11.15 ± 1.83 mm, respectively. While the same distances for the female edentulous group were 11.42 ± 2.95, 11.50 ± 2.83, and 10.64 ± 2.06 mm, respectively. The grouped average for the distance between the first, second, and third molars and the lingual nerve for the edentulous group was 13.81 ± 3.42, 13.20 ± 3.04, and 10.90 ± 1.94 mm.

**Table 2:** The average distances between the first, second, and third mandibular molar and the lingual nerve.

Statistics	Average distance between the third mandibular molar to the lingual nerve (mm)	Average distance between the second mandibular molar to the lingual nerve (mm)	Average distance between the first mandibular molar to the lingual nerve (mm)
Dentulous			
Female	11.46 ± 2.51	15.5 ± 5.24	18.75 ± 5.91
Male	10.98 ± 1.27	15.75 ± 2.61	19.65 ± 4.50
Average	11.22 ± 1.89	15.63 ± 3.93	19.20 ± 5.21
Edentulous			
Female	10.64 ± 2.06	11.50 ± 2.83	11.42 ± 2.95
Male	11.15 ± 1.83	14.90 ± 3.25	16.20 ± 3.88
Average	10.90 ± 1.94	13.20 ± 3.04	13.81 ± 3.42

All left and right lingual nerves are bifurcated above the mandibular notch. The LN's course superior or inferior to the submandibular duct (SMD), the bifurcation point of the LN from the mandibular division of the trigeminal nerve, as well as the branching pattern and number of LN branches is given (Table 3). The results for dentulous males and females and edentulous males and females were pooled separately. The lingual nerve crossed above the SMD in 52.17% of cases on the right and 26.09% on the left, while it passed below the SMD in 47.83% on the right and 73.91% on the left. All lingual nerves are bifurcated above the mandibular notch. On the right, the nerve had two branches in 4.17%, three in 41.67%, four in 20.83%, five in 25%, and seven in 2.33% of cases. On the left, there were three branches in 45.83%, four in 20.83%, five in 16.67%, six in 4.17%, seven in 8.33%, and nine in 4.17%. The right lingual nerve was classified as 66.67% Type 1, 29.17% Type 2, and 4.17% Type 3, while the left was 80% Type 1 and 20% Type 2.

**Table 3:** Data of the lingual nerve's (LN) relationship to the submandibular duct, the location of the bifurcation point, the number of branches entering the tongue, and the classification of the main trunk of the lingual nerve branches.

Category	Right (n)	Left (n)	Total (n)
LN relation to the submandibular duct			
Superior	12 (52.17%)	6 (26.09%)	18 (39.13%)
Inferior	11 (47.83%)	17 (73.91%)	28 (60.87%)
Total	23	23	46
LN bifurcation point			
Above mandibular notch	24 (100%)	25 (100%)	49 (100%)
Total	24	25	49
Branches of LN entering the tongue			
Two	1 (4.17%)	—	1 (2.08%)
Three	10 (41.67%)	11 (45.83%)	21 (43.75%)
Four	5 (20.83%)	5 (20.83%)	10 (20.83%)
Five	6 (25.00%)	4 (16.67%)	10 (20.83%)
Six	—	1 (4.17%)	1 (2.08%)
Seven	2 (8.33%)	2 (8.33%)	4 (8.33%)
Nine	—	1 (4.17%)	1 (2.08%)
Total	24	24	48
Classification of the main trunk of the LN branches			
Type 1	16 (66.67%)	20 (80%)	36 (73.33%)
Type 2	7 (29.17%)	5 (20%)	12 (24.58%)
Type 3	1 (4.17%)	—	1 (2.08%)
Total	24	25	49

*Note:* The number of branches is the percentage of the respective row divided by that section's respective total.

Abbreviation: LN = lingual nerve.

To compare the left and right sides from Table 3 for the lingual nerve (LN) relation to the submandibular duct (SMD), McNemar's test was applied, and the *p*‐value was 0.03125. This indicates a statistically significant difference between the left and right sides in the relationship of the lingual nerve to the submandibular duct at the 5% significance level.

## 4 Discussion

Temporary LN damage varies from 0% to 37%, while permanent LN injury during third molar surgeries ranges from 0.02% to 2% (Tojyo et al.2019). In this study, the distance from the alveolar ridge near the third molar to the lingual nerve in dentulous samples closely resembled the distances reported by Ostrowski et al. (2023). Ostrowski et al. (2023) had the distances between the lingual nerve and the first, second, and third molar as 19.81 ± 5.41, 14.62 ± 3.22, and 11.59 ± 2.12 mm, respectively. In the edentulous group, small differences compared to the dentulous sample were noted. This may be attributed to bone reabsorption, likely influenced by the advanced age of the sample (Ostrowski et al. 2023). There was a difference between the distances recorded by this study compared to Mendes et al. (2013)*;* however, this may be due to the different population samples and the age of the cadavers (Ostrowski et al. 2023). This study, Ostrowski et al. (2023) and Chalise et al. (2022) had a millimeter difference for the dentulous population (Ostrowski et al. 2023; Chalise et al. 2022). The male dentulous population differences may be due to the Nepalese population compared to the white South African population and the sample size (Chalise et al. 2022). In the South African sample, the distances between the first, second, and third molars and lingual nerve were larger in males compared to females. This is possibly due to sexual dimorphism in the South African population, where females have smaller mandibular measurements (Steyn and İşcan 1998).

The measurements from the research conducted by Chan et al. (2010) for the first and second molars were 13 and 9.6 mm, respectively. Chan et al. (2010) investigated a United States population, potentially incorporating diverse subpopulations in a single group, with a notable age range (Chan et al. 2010). In this study, the distance between the LN and the alveolar ridge of the second molar in the dentulous sample was slightly larger than the study by Ostrowski et al. (2023). In this study, the vertical distance between the LN and the alveolar ridge of the first molar in the dentulous sample was similar to the distances found by Ostrowski et al. (2023).

Ostrowski et al. (2023) did not specify molar presence in their study, while Mendes et al. (2013) noted the presence or absence of third molars (Ostrowski et al. 2023; Mendes, de Carvalho Leite Leal Nunes, and de Almeida Lopes 2013). Chalise et al. (2022) exclusively used cadavers with second and third molars present, and Chan et al. (2010) excluded six unilateral edentulous molars from their study. In our study, both dentulous and edentulous cadavers were included, which is relatively uncommon in the existing literature when exploring the relationship between the LN and the alveolar ridge.

The study results show that the distance between the lingual nerve and the third molar is the smallest of the three molar measurements. This is an important observation, as surgery is common in this region. Our study shows similar results for the vertical distance between the lingual nerve and third molar for dentulous cadaver studies by Erdogmus et al. (2008), Pogrel et al. (1995), Kieselbach and Chamberlain (1984), Behnia et al. (2000), Hὄlzle and Wolff (2007), Karakas et al. (2007), and Dias et al. (2015).

Notably, there is a scarcity of research investigating edentulous patients, with limited studies such as those conducted by Shimoo et al. (2017). There are significant alveolar changes between dentulous and edentulous bone; however, the differences between dentulous and edentulous mandibles have received minimal attention (Shimoo et al. 2017).

Methodologically, Ostrowski et al. (2023) used cadavers, MRI, ultrasound, and surgical techniques for data collection. Mendes et al. (2013) dissected cadavers following sagittal dissection of the heads. Chan et al. (2010) reported dissection of the lingual nerve, and Chalise et al. (2022) exposed the lingual nerve through infra‐temporal dissection. In our study, we hemisected each cadaver head and dissected, and measured the lingual nerve on the lateral surface of the molar region. The different methods may account for different measurements found between the molars and lingual nerve.

In our investigation, most lingual nerves crossed the SMD superiorly on the right side. On the left side, the majority of LNs crossed the SMD inferiorly. The right side in this study differs from the study by Mendes et al. (2013), Iwai et al. (2022), and Ostrowski et al. (2023), which may be a result of the sample size and the population. The left‐sided samples were similar to the existing literature (Ostrowski et al. 2023; Mendes, de Carvalho Leite Leal Nunes, and de Almeida Lopes 2013; Iwai et al. 2022). Iwai et al. (2022) found that in 33 patients, the lingual nerve crosses below the SMD equally on the right and left side. A reason for this difference is possibly due to population differences. A study by Günenç Beşer et al. (2018) reported similar findings, where the lingual nerve crossed superior to the SMD. Surgeons should be aware of this anatomical variation to minimize the risk of injury to the lingual nerve or the SMD during procedures.

The bifurcation point in this study was found to be above the mandibular notch in all the samples. Kim et al. (2003) defined four bifurcation points of the lingual nerve, namely, Type 1, where the lingual nerve bifurcated above the mandibular notch, Type 2, where the lingual nerve bifurcated in the upper half between the mandibular notch and mandibular lingula, Type 3, where the lingual nerve bifurcated in the lower half between the mandibular notch and mandibular lingula, and Type 4, where the mandibular nerve branches in a plexiform (Kim et al. 2003). In Ostrowski et al. (2023), the majority of the LN bifurcation was above the mandibular notch, and the second most found location was in the upper half between the mandibular notch and the mandibular lingula. The difference in the bifurcation pattern may be explained by several factors, such as the difference in sample size and varied population groups, which consisted of populations from all over the world except for Africa. In a study by Edrogmus et al. (2008), the lingual nerve bifurcated above the mandibular notch in 66.7% of the sample; this was classified as a Type 1 bifurcation. Thus, 100% of our sample has a Type 1 bifurcation of the lingual nerve.

The number of terminal branches that enter the tongue is important when performing a lingual nerve block to remove the mandibular third molar (Chan et al. 2010; Pogrel et al. 1995; Iwanaga et al. 2028; Balasubramanian et al. 2017; Pippi, Spota, and Santoro 2017; Garisto et al. 2010). In this study, the variations in pattern prevalence were notably different from Al‐Amery et al. (2016), who identified four distinct patterns. In this study, Pattern 4 was found to be the most prevalent according to the classification system that was used by Al‐Amery et al. (2016). In this study, the maximum number of terminal branches for the lingual nerve was nine on the right side in one cadaver, while on the left side, there were seven terminal branches. A different classification system may be required as pattern 4 included too many different terminal branches of the LN. Pattern 4 included four or more terminal branches which is why this study's percentage was different from Al‐Amery et al. (2016), since this study had a greater number of terminal branches. Al‐Amery et al. (2016) found the two terminal branches in their sample as the most common. Rusu et al. (2008) found a different pattern of insertion of the terminal branches of the lingual nerve when compared to the study by Al‐Amery et al. (2016). Rusu et al. (2008) only distinguished between two morphological types of terminal branches. Fagan and Roy (2024) recognized four terminal branches entering the tongue. Five terminal branches were found in a study by Zur et al. (2004). According to Zur et al. (2004), these branches innervate the lingual mucosa, interconnect with the hypoglossal nerve, and provide bilateral innervation to the tongue. The difference between our study and those by Al‐Amery (2016) and Rusu et al. (2008) may be attributed to population differences and the samples being studied and may differ between the left and right side in the same cadaver.

Regarding the collateral branches of the lingual nerve, Shimotakahara et al. (2019) reported that the Type 1 branching pattern was the most common, followed by Type 2, with Type 3 being the least frequent (Shimotakahara et al. 2019). Our study observed a similar branching pattern to Shimotakahara et al. (2019), with slight variations that could be attributed to differences in sample size and the specific populations studied, given the contrast between the Japanese population in their research and our white South African population.

In conclusion, our study on the lingual nerve in a white South African population provided valuable insights into its anatomical characteristics during dental procedures. Notably, differences were observed in measurements and patterns compared to the existing literature and other populations, highlighting the need for further research. Comparisons between the right and left lingual nerves were done. The study emphasizes the importance of considering diverse populations, specifically the black South African population, to enhance the generalizability of findings for third‐molar surgeries. The consideration of edentulous cases and the use of hemi‐secting the head for improved access to the oral cavity are some of the strengths of this study. Overall, this study underscores the need for continued research to refine our understanding of lingual nerve anatomy and improve surgical outcomes.

The data collected will help to minimize the risk of iatrogenic damage during dental procedures.

### 4.1 Limitations of the Study

The cadavers in the study were of advanced age, with 14 being edentulous. The study exclusively focused on the white South African population. It is recommended to incorporate cadavers from the black South African population to enhance diversity and increase the sample size. Additionally, including a younger population undergoing third molar surgeries with the aid of ultrasound is crucial for a more comprehensive analysis.

## Author Contributions

Conceptualization: A. Uys. Methodology: A. Uys, A. Procos, and R. Human‐Baron. Validation: A. Uys and R. Human‐Baron. Formal analysis: A. Uys and A. Procos. Investigation: A. Procos. Resources: A. Uys and R. Human‐Baron. Data Curation: A. Uys and A. Procos. Writing–original draft: A. Uys, R. Human‐Baron, and A. Procos. Writing–review and editing: A. Uys, R. Human‐Baron, and A. Procos. Supervision: A. Uys and R. Human‐Baron. Project administration: A. Uys.

## Ethics Statement

Ethical clearance for the use of cadaver tissue was obtained from the Faculty of Health Sciences Research Ethics Committee (185/2023). All dissections, measurements, and observations were in accordance with the Declaration of Helsinki.

## Conflicts of Interest

The authors declare no conflicts of interest.

## Data Availability

Data that support the findings of this study are available from the corresponding author upon reasonable request. All data is contained within the manuscript.

## References

[cre270051-bib-0001] Al‐Amery, S. M. , P. Nambiar , M. Naidu , and W. C. Ngeow . 2016. “Variation in Lingual Nerve Course: A Human Cadaveric Study.” PLoS One 11, no. 9: e0162773. 10.1371/journal.pone.0162773. 27662622 PMC5035068

[cre270051-bib-0002] Bagheri, S. C. , R. A. Meyer , H. A. Khan , A. Kuhmichel , and M. B. Steed . 2010. “Retrospective Review of Microsurgical Repair of 222 Lingual Nerve Injuries.” Journal of Oral and Maxillofacial Surgery 68, no. 4: 715–723. 10.1016/j.joms.2009.09.111. 20036042

[cre270051-bib-0003] Balasubramanian, S. , E. Paneerselvam , T. Guruprasad , M. Pathumai , S. Abraham , and V. Krishnakumar Raja . 2017. “Efficacy of Exclusive Lingual Nerve Block Versus Conventional Inferior Alveolar Nerve Block in Achieving Lingual Soft‐Tissue Anesthesia.” Annals of Maxillofacial Surgery 7, no. 2: 250–255. 10.4103/ams.ams6517. 29264294 PMC5717903

[cre270051-bib-0004] Behnia, H. , A. Kheradvar , and M. Shahrokhi . 2000. “An Anatomic Study of the Lingual Nerve in the Third Molar Region.” Journal of Oral and Maxillofacial Surgery 58, no. 6: 649–651. 10.1016/s0278-2391(00)90159-9. 10847287

[cre270051-bib-0005] Chalise, U. , R. Kharbuja , S. Dhungel , and K. Bimb . 2022. “Lingual Nerve in Relation to Mandibular Third Molar Region: A Cadaveric Study.” Journal of Chitwan Medical College 12, no. 1: 98–101. 10.54530/jcmc.659.

[cre270051-bib-0006] Chan, H. L. , D. J. M. Leong , J. H. Fu , C. Y. Yeh , N. Tatarakis , and H. L. Wang . 2010. “The Significance of the Lingual Nerve During Periodontal/Implant Surgery.” Journal of Periodontology 81, no. 3: 372–377. 10.1902/jop.2009.090506. 20192863

[cre270051-bib-0007] Dias, G. J. , R. K. de Silva , T. Shah , et al. 2015. “Multivariate Assessment of Site of Lingual Nerve.” British Journal of Oral and Maxillofacial Surgery 53, no. 4: 347–351. 10.1016/j.bjoms.2015.01.011. 25662169

[cre270051-bib-0008] Erdogmus, S. , F. Govsa , and S. Celik . 2008. “Anatomic Position of the Lingual Nerve in the Mandibular Third Molar Region as Potential Risk Factors for Nerve Palsy.” Journal of Craniofacial Surgery 19, no. 1: 264–270. 10.1097/scs.0b013e31815c9411. 18216699

[cre270051-bib-0009] Fagan, S. E. , and W. Roy . 2024. Antomy, Head and Neck, Lingual Nerve (2023). Treasure Island, FL: StatPearls Publishing. PMID: 31536258 Bookshelf ID: NBK546652. 31536258

[cre270051-bib-0010] Garisto, G. A. , A. S. Gaffen , H. P. Lawrence , H. C. Tenenbaum , and D. A. Haas . 2010. “Occurrence of Paresthesia After Dental Local Anesthetic Administration in the United States.” Journal of the American Dental Association 141: 836–844. 10.14219/jada.archive.2010.0281. 20592403

[cre270051-bib-0011] Graff‐Radford, S. B. , and R. W. Evans . 2003. “Lingual Nerve Injury.” Headache 43, no. 9: 975–983. 10.1046/j.1526-4610.2003.03189.x. 14511274

[cre270051-bib-0012] Günenç Beşer, C. , B. Erçakmak , H. B. Ilgaz , A. Vatansever , and M. F. Sargon . 2018. “Revisiting the Relationship Between the Submandibular Duct, Lingual Nerve and Hypoglossal Nerve.” Folia Morphologica 77, no. 3: 521–526. 10.5603/FM.a2018.0010. 29399751

[cre270051-bib-0013] Hölzle, F. W. , and K. D. Wolff . 2001. “Anatomic Position of the Lingual Nerve in the Mandibular Third Molar Region With Special Consideration of an Atrophied Mandibular Crest: An Anatomical Study.” International Journal of Oral and Maxillofacial Surgery 30: 333–338. 10.1054/ijom.2001.0064. 11518358

[cre270051-bib-0014] Iwai, T. , S. Sugiyama , S. Oguri , and K. Mitsudo . 2022. “Anatomical Relationship Between the Lingual Nerve and Submandibular Duct.” Journal of Craniofacial Surgery 33, no. 3: 949–950. 10.1097/SCS.0000000000008168. 34538801

[cre270051-bib-0015] Iwanaga, J. , P. J. Choi , M. Vetter , et al. 2028. “Anatomical Study of the Lingual Nerve and Inferior Alveolar Nerve in the Pterygomandibular Space: Complications of the Inferior Alveolar Nerve Block.” Cureus 10, no. 8: e3109. 10.7759/cureus.3109. PMC6175254 30338184

[cre270051-bib-0016] Karakas, P. , M. Üzel , and J. Koebke . 2007. “The Relationship of the Lingual Nerve to the Third Molar Region Using Radiographic Imaging.” British Dental Journal 203, no. 1: 29–31. 10.1038/bdj.2007.584. 17632483

[cre270051-bib-0017] Kiesselbach, J. E. , and J. G. Chamberlain . 1984. “Clinical and Anatomic Observations on the Relationship of the Lingual Nerve to the Mandibular Third Molar Region.” Journal of Oral and Maxillofacial Surgery 42: 565–567. 10.1016/0278-2391(84)90085-5. 6590806

[cre270051-bib-0018] Kim, H. J. , S. Y. Kim , K. S. Hu , I. H. Chung , and E. W. Lee . 2004. “Topographic Anatomy of the Lingual Nerve and Variations in Communication Pattern of the Mandibular Nerve Branches.” Surgical and Radiologic Anatomy 26, no. 2: 128–135. 10.1007/s00276-003-0179-x. 14586562

[cre270051-bib-0019] Kushnerev, E. , and J. M. Yates . 2015. “Evidence‐Based Outcomes Following Inferior Alveolar and Lingual Nerve Injury and Repair: A Systematic Review.” Journal of Oral Rehabilitation 42, no. 10: 786–802. 10.1111/joor.12313. 26059454

[cre270051-bib-0020] Mendes, M. B. M. , C. M. de Carvalho Leite Leal Nunes , and M. C. de Almeida Lopes . 2013. “Anatomical Relationship of Lingual Nerve to the Region of Mandibular Third Molar.” Journal of Oral and Maxillofacial Research 4, no. 4: e2. 10.5037/jomr.2013.4402. PMC3904728 24478912

[cre270051-bib-0021] Ostrowski, P. , M. Bonczar , J. Wilk , et al. 2023. “The Complete Anatomy of the Lingual Nerve: A Meta‐Analysis With Implications for Oral and Maxillofacial Surgery.” Clinical Anatomy 36, no. 6: 905–914. 10.1002/ca.24033. 36864652

[cre270051-bib-0022] Piagkou, M. , T. Demesticha , P. Skandalakis , and E. O. Johnson . 2011. “Functional Anatomy of the Mandibular Nerve: Consequences of Nerve Injury and Entrapment.” Clinical Anatomy 24, no. 2: 143–150. 10.1002/ca.21089. 21322036

[cre270051-bib-0023] Pichler, J. W. , and O. R. Beirne . 2001. “Lingual Flap Retraction and Prevention of Lingual Nerve Damage Associated With Third Molar Surgery: A Systematic Review of the Literature.” Oral Surgery, Oral Medicine, Oral Pathology, Oral Radiology, and Endodontology 91, no. 4: 395–401. 10.1067/moe.2001.114154. 11312457

[cre270051-bib-0024] Pippi, R. , A. Spota , and M. Santoro . 2017. “Prevention of Lingual Nerve Injury in Third Molar Surgery: Literature Review.” Journal of Oral and Maxillofacial Surgery 75: 890–900. 10.1016/j.joms.2016.12.040. 28142010

[cre270051-bib-0025] Pogrel, M. A. , A. Renaut , B. Schmidt , and A. Ammar . 1995. “The Relationship of the Lingual Nerve to the Mandibular Third Molar Region: An Anatomic Study.” Journal of Oral and Maxillofacial Surgery 53, no. 10: 1178–1181. 10.1016/0278-2391(95)90630-4. 7562172

[cre270051-bib-0026] Rusu, M. C. , V. Nimigean , L. Podoleanu , R. V. Ivaşcu , and M. C. Niculescu . 2008. “Details of the Intralingual Topography and Morphology of the Lingual Nerve.” International Journal of Oral and Maxillofacial Surgery 37, no. 9: 835–839. 10.1016/j.ijom.2008.05.014. 18599272

[cre270051-bib-0027] Shimoo, Y. , M. Yamamoto , M. Suzuki , et al. 2017. “Anatomic and Histological Study of Lingual Nerve and Its Clinical Implications.” Bulletin of Tokyo Dental College 58, no. 2: 95–101. 10.2209/tdcpublication.2016-0010. 28724864

[cre270051-bib-0028] Shimotakahara, R. , H. Lee , K. Mine , S. Ogata , and Y. Tamatsu . 2019. “Anatomy of the Lingual Nerve: Application to Oral Surgery.” Clinical Anatomy 32, no. 5: 635–641. 10.1002/ca.23361. 30815909

[cre270051-bib-0029] Shinohara, H. , I. Mataga , and I. Kageyama . 2010. “Discussion of Clinical Anatomy of the Lingual Nerves.” Okajimas Folia Anatomica Japonica 87, no. 3: 97–102. 10.2535/ofaj.87.97. 21174938

[cre270051-bib-0030] Sittitavornwong, S. , M. Babston , D. Denson , S. Zehren , and J. Friend . 2017. “Clinical Anatomy of the Lingual Nerve: A Review.” Journal of Oral and Maxillofacial Surgery 75, no. 5: 926.e1–926.e9. 10.1016/j.joms.2017.01.009. 28189657

[cre270051-bib-0031] Steyn, M. , and M. Y. İşcan . 1998. “Sexual Dimorphism in the Crania and Mandibles of South African Whites.” Forensic Science International 98, no. 1–2: 9–16. 10.1016/s0379-0738(98)00120-0. 10036755

[cre270051-bib-0032] Tojyo, I. , T. Nakanishi , Y. Shintani , K. Okamoto , Y. Hiraishi , and S. Fujita . 2019. “Risk of Lingual Nerve Injuries in Removal of Mandibular Third Molars: A Retrospective Case‐Control Study.” Maxillofacial Plastic and Reconstructive Surgery 41, no. 1: 40. 10.1186/s40902-019-0222-4. 31555619 PMC6733934

[cre270051-bib-0033] Touré, G. , L. Bicchieray , J. Selva , and C. Vacher . 2005. “The Intra‐Lingual Course of the Nerves of the Tongue.” Surgical and Radiologic Anatomy 27, no. 4: 297–302. 10.1007/s00276-005-0335-6. 16244780

[cre270051-bib-0034] Zur, K. B. , L. Mu , and I. Sanders . 2004. “Distribution Pattern of the Human Lingual Nerve.” Clinical Anatomy 17, no. 2: 88–92. 10.1002/ca.10166. 14974094

